# Biomarker-based approach to determine etiology and severity of pulmonary hypertension: Focus on microRNA

**DOI:** 10.3389/fcvm.2022.980718

**Published:** 2022-10-06

**Authors:** Sylwester Rogula, Bartosz Pomirski, Norbert Czyżak, Ceren Eyileten, Marek Postuła, Łukasz Szarpak, Krzysztof J. Filipiak, Marcin Kurzyna, Miłosz Jaguszewski, Tomasz Mazurek, Marcin Grabowski, Aleksandra Gąsecka

**Affiliations:** ^1^1st Chair and Department of Cardiology, Medical University of Warsaw, Warsaw, Poland; ^2^Department of Experimental and Clinical Pharmacology, Centre for Preclinical Research and Technology, Medical University of Warsaw, Warsaw, Poland; ^3^Genomics Core Facility, Center of New Technologies (CeNT), University of Warsaw, Warsaw, Poland; ^4^Department of Outcomes Research, Maria Skłodowska-Curie Medical Academy in Warsaw, Warsaw, Poland; ^5^Institute of Clinical Sciences, Maria Skłodowska-Curie Medical Academy in Warsaw, Warsaw, Poland; ^6^Department of Pulmonary Circulation, Thromboembolic Diseases and Cardiology, Centre of Postgraduate Medical Education, European Health Centre Otwock, Otwock, Poland; ^7^1st Department of Cardiology, Medical University of Gdańsk, Gdańsk, Poland

**Keywords:** microRNA, miRNA, biomarker, pulmonary arterial hypertension, PAH

## Abstract

Pulmonary arterial hypertension (PAH) is characterized by remodeling of the pulmonary arteries, and defined by elevated pulmonary arterial pressure, measured during right heart catheterization. There are three main challenges to the diagnostic and therapeutic process of patients with PAH. First, it is difficult to differentiate particular PAH etiology. Second, invasive diagnostic is required to precisely determine the severity of PAH, and thus to qualify patients for an appropriate treatment. Third, the results of treatment of PAH are unpredictable and remain unsatisfactory. MicroRNAs (miRNAs) are small non-coding RNAs that regulate post transcriptional gene-expression. Their role as a prognostic, and diagnostic biomarkers in many different diseases have been studied in recent years. MiRNAs are promising novel biomarkers in PAH due to their activity in various molecular pathways and processes underlying PAH. Lack of biomarkers to differentiate between particular PAH etiology and evaluate the severity of PAH, as well as paucity of therapeutic targets in PAH open a new field for the possibility to use miRNAs in these applications. In our article, we discuss the potential of miRNAs use as diagnostic tools, prognostic biomarkers and therapeutic targets in PAH.

## Introduction

PAH is a heterogeneous disease driven most often by pathogenic remodeling of distal pulmonary arterioles and an increase in pulmonary vascular resistance (1). PAH is a rare disease, with the prevalence and incidence of 15–60 subjects per million population and 5–10 cases per million per year (2). Patients with PAH often have severe and progressive disease. Symptoms of PAH are divided by the WHO into four functional classes, graded according to the severity of the symptoms, with class I describing the mildest and class IV the most severe PAH. This classification helps the clinicians to assess patients’ functional status and determine the appropriate treatment.

### Three main challenges in pulmonary arterial hypertension

Recently, the current knowledge regarding the general miRNA expression changes in PAH has been thoroughly summarized (3). In this article, we have focused on three clinical problems in patients with PAH, which could be solved using miRNA-based biomarkers.

Firstly, differentiation of the etiology of PAH is difficult. In registries, about 50% of patients with PAH have idiopathic, heritable or drug-induced PAH. It is important to increase our knowledge in order to minimize the diagnosis of idiopathic PAH. The remaining cases of PAH, described as associated PAH, can be caused by various conditions, such as connective tissue disease (CTD), human immunodeficiency virus (HIV) infection, schistosomiasis or portal hypertension (4). The leading cause in the subgroup of associated PAH is CTD, mainly systemic sclerosis (5). In systemic sclerosis, it is difficult to determine whether increased pressure in pulmonary artery is caused by pulmonary fibrosis (ESC group 3), small vessel disease (ESC group 1 = PAH) or left heart failure associated with left ventricle diastolic dysfunction (ESC group 2). Differentiation of the cause is extremely important due to the different treatment. Congenital heart disease (CHD) can also play a role in the development of PAH. It is estimated, that 10% of adults with CHD may also have PAH (6). Currently, there is no specific blood-based biomarker for PAH, although numerous markers have been tested (7), such as inflammatory markers [growth differentiation factor-15 (8), osteopontin (9), neutrophil to lymphocyte ratio (10), stem cell growth factor β (11)], heart function markers (B-type natriuretic peptide, amino-terminal pro-B type natriuretic peptide or red cell distribution width) or oxidative stress related biomarkers [F2-isoprostanes (12) or oxidized lipids] (9, 13).

Second, it is challenging to determine the severity of PAH, and thus to qualify patients for an appropriate treatment. Although some blood test might help to identify the PAH etiology, they are not useful in diagnosing or assessing the clinical advancement of the disease (7). Hence, the diagnostic workup currently includes repeated RHC, which is an invasive procedure with a risk of complications estimated according at about 1%. Altogether, PAH requires novel tools to evaluate the clinical severity of the disease.

Third, the results of PAH treatment remain unsatisfactory (7). As stated in the guidelines of the European Society of Cardiology, there are three pathways underlying the pathogenesis of PAH: the endothelin, nitric oxide (NO) and prostacyclin (PGI_2_) pathways (7). Even though treatments targeting each one of them, including endothelin receptor antagonists (ERA), phosphodiesterase type 5 inhibitors (PDE-5) and PGI_2_ analogs are well established in clinical practice, the functional limitation and survival of patients leaves room for substantial improvement (7). Hence, new therapeutic targets in PAH are urgently needed.

### Potential of microRNA

MicroRNAs(miRNAs) are small non-coding RNAs that regulate post transcriptional gene-expression. MiRNAs can induce mRNA degradation and translational repression by interacting with the 3′ untranslated region (3′ UTR) of target mRNAs, but their interactions with other regions of mRNAs have also been reported. In some circumstances, they can also activate translation or regulate transcription. However, miRNA interaction with their target genes is very dynamic and dependent on many factors, such as subcellular location of miRNAs, the abundancy of miRNAs and target mRNAs, and the affinity of miRNA-mRNA interactions (14). MiRNAs are found in all eukaryotic cells across the species. Over the last few years, their role in the progression and development of numerous diseases has been extensively studied, since they hold enormous potential for prognostic, and diagnostic biomarkers as well as therapeutics (15–19). Studies showed that miRNAs are also promising novel biomarkers in PAH due to their activity in various molecular pathways and processes underlying PAH (3, 20). The lack of biomarkers in PAH enabling the differentiation of individual PAH etiologies and the assessment of the severity of PAH as well as the shortage of therapeutic targets in PAH opens a new field for the possibility of using miRNAs in these applications (3).

In this review, we present current challenges in miRNA analysis and interpretation in PAH and summarize the steps which need to be taken before clinical applicability of miRNAs in PAH.

## Pathophysiology of pulmonary arterial hypertension

Pathophysiology of PAH may differ depending on the cause of the disease, but in general, it is associated with vascular abnormality. PAH is a progressive disease, causing the narrowing of the blood vessels in the lungs, which results in a resistance to blood flow and an increase in pulmonary artery pressures (21). In addition to that, PAH may be also attributed to lesions occurring in distal muscular-type arteries, ranging in diameter from 500 μm down to even 70 μm in humans (22). The underlying mechanisms involve impaired function of signaling pathways of NO, PGI_2_, thromboxane A_2_ (TXA_2_), and endothelin-1 (ET-1) (23), which leads to a vasoconstrictive effect of an upregulated ET-1, with concurrent impairment of vasodilation caused by reduced PGI_2_ production and NO synthase function (24). Altogether, these changes lead to an increase in pulmonary vascular resistance (25). The progressive increase in right ventricular (RV) afterload leads to RV hypertrophy and, ultimately, RV failure and death (26).

Moreover, there are differences in the miRNA concentrations between animals with RV hypertrophy and RV failure, which suggests specific changes in the signature of miRNAs during PAH progression (27, 28). The mechanisms underlying the transition from compensated RV hypertrophy to RV failure remain unclear, and further investigations are needed to gain a better understanding of miRNA expression patterns in RV pathological remodeling (28, 29).

## Key microRNAs associated with pulmonary arterial hypertension

Numerous miRNAs participate in the pathogenesis of PAH, some of them being upregulated, and some other downregulated in PAH lung samples compared to normal controls (20). The expression of many miRNAs in patients with PAH, and healthy control subjects in different tissues was examined. Many of these miRNAs were characterized by distinct expression pattern in human PAH lungs and in animal PAH models, although 4 miRNAs turned out to have similar expression pattern among the different models, making them the key miRNAs for the potential use in diagnostic and therapeutic processes. These four highlighted miRNAs were miR-29, miR-124, miR-140, and miR-204 (3).

MiR-204, as a representative of this group, is a muscle-specific miRNA that is highly active in cardiomyocytes and vascular muscle cells (30). MiR-204 has been found to be downregulated in diseased pulmonary arterial smooth muscle cells (PASMCs) in PAH and is linked to signaling molecules crucial to PAH including PPAR-γ, TGF-β1, STAT3, SHP2, BRD4, NFAT, and HIF-1α (20, 30, 31). Mir-204 may also mediate the protective action of mesenchymal stromal cell-derived exosomes (32) and altered levels correlate with more angiogenic and proliferative disease (33) in both the pulmonary and coronary circulation (34).

Moreover, the specific miRNAs grouped into clusters might be associated with particular mechanisms of PAH pathogenesis. MiRNAs associated with PASMCs proliferation, migration and contraction were the miR-17-92 cluster, miR-21, miR-124, miR-143/145 cluster, miR-204, and miR-210. Those associated with pulmonary artery endothelial homeostasis in PAH were the miR-17-92 cluster, miR-21, miR-27a, miR-424, and miR-503. MiRNA reported to regulate fibroblasts was miR-124 (20). Reduced miR-124 causes hyperproliferation and migration of fibroblasts, whereas overexpression of miR-124 inhibits proliferation and migration of fibroblasts (35). A list of dysregulated miRNAs in PAH are presented in Table 1.

**TABLE 1:** A list of dysregulated miRNAs in PAH.

MicroRNA	Changes in PAH	Sample type	Targets	Function	Methodology	References
miR-17–92	↓	Human PASMCs	*PDLIM5*	PASMC differentiation	qRT-PCR	(20, 59)
	Transient ↑	Hypoxic PAH mouse lung homogenates	p21	PASMC proliferation	Microarray, qRT-PCR	(47)
miR-145	↑	Human HPAH and IPAH lung tissue and PASMCs	*KLF4, SMAD4, SMAD5*	PASMC differentiation and inhibition of PASMC proliferation	Northern blot, qRT-PCR	(3, 96)
		Hypoxic PAH mouse PASMCs				
		*BMPR2* mutation PAH PASMCs				
miR-21	↑	Human PAH PASMCs	*PTEN*	PASMC proliferation	qRT-PCR	(97)
	↑	Human PAH PAECs	*BMPR2, RhoB*	Decrease angiogenesis and vasodilatation	Network based analysis, qRT-PCR	(98)
miR-204	↓	Human PAH PASMCs	*SHP2, HIF-1*α	Apoptosis and inhibition of PASMC proliferation	qRT-PCR	(99)
		Human PAH lung biopsies				
		Hypoxic PAH mouse lung homogenates				
miR-424/miR-503	↓	Human plasma	*SMURF1, BMPR2*	RV hypertrophy	qRT-PCR	(100)
miR-124	↓	Human PAH fibroblasts	Monocyte chemotactic protein-1 and polypyrimidine tract-binding protein 1	Antiproliferation of fibroblasts	qRT-PCR	(35)
miR-27a	↑	Hereditary PAH PAECs PASMCs	*BMPR2, SMAD9*	Antiproliferation	qRT-PCR	(101)
miR-29	↓	Hypoxic PAH rat PAAFs	α-SMA, extracellular matrix collagen	Fibrosis	qRT-PCR	(102)
miR-140	↑	Human hypoxia PASMCs	*SOD2*	PASMC proliferation	qRT-PCR	(103)
miR-210	↑	Human PASMCs	*E2F3*	Hyperplasia of PASMCs,	Microarray, qRT-PCR	(104)

HPAH, hereditary pulmonary arterial hypertension; IPAH, idiopathic pulmonary arterial hypertension; MCT, monocrotaline; NA, not available; PAEC, pulmonary artery endothelial cell; PAH, pulmonary arterial hypertension; PASMC, pulmonary artery smooth muscle cell; qRT-PCR, quantitative real-time polymerase chain reaction.

## Circulating microRNA as diagnostic and prognostic biomarkers

Studies suggest that miRNAs can be used as stable blood-based markers for many medical conditions. They were first established as biomarkers for cancer in 2008 when expression levels of tumor-associated miRNAs in serum from diffuse large B-cell lymphoma (DLBCL) patients was compared with healthy controls (36). Since then, their potential use as biomarkers has been repeatedly proposed for various diseases. The advantage of miRNAs as biomarkers also results from the fact, that miRNAs are present in numerous body fluids. The existence of miRNAs in 12 body fluids and urine from patients with various conditions were examined. The results showed the presence of miRNAs in all the body fluid types tested and different compositions in different fluid types. Studies also showed different miRNA patterns in the urine samples from patients with distinct conditions (37), which gives hope that getting to know specific miRNAs expression patterns in particular body fluids can help evaluate crucial information about current condition of the patients, such as severity of the disease or assessing a particular etiology of the disease.

Another advantage of miRNAs is that they are expressed in stable biomolecules, such as extracellular vesicles, RNA-lipid complexes or RNA–protein complexes (38, 39). Plasma miRNAs were found to be remarkably stable even under conditions as harsh as boiling, low or high pH, long-time storage at room temperature, and multiple freeze-thaw cycles (40, 41).

The promising results of the previous studies has encouraged to look for diagnostic roles of miRNAs in PAH. As it turned out, they can be used as biomarkers in two ways: (i) miRNA differentiates the type of PAH; (ii) miRNA differentiates the severity of PAH (31, 42).

### MicroRNA to differentiate the type of pulmonary arterial hypertension

**FIGURE 1:**
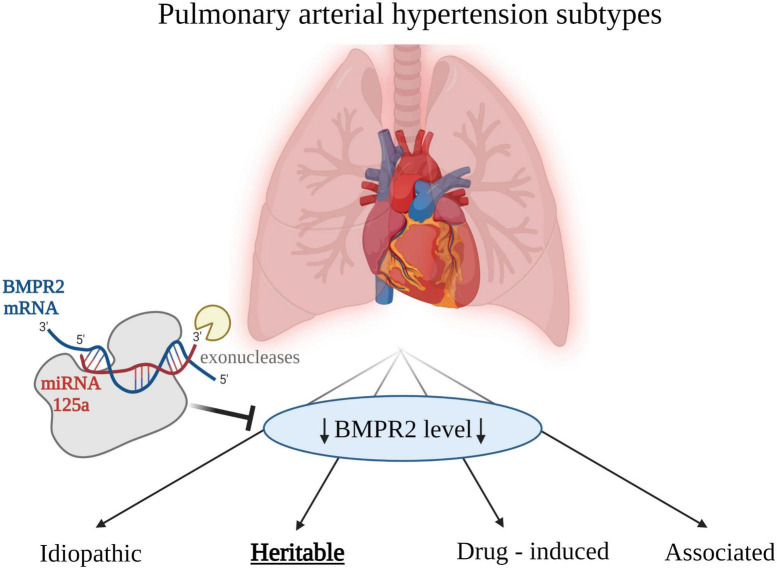
Etiologies of pulmonary arterial hypertension (PAH). Low level of bone morphogenetic protein receptor type 2 (BMPR2) is associated with PAH in general, however the association is strongest for the heritable PAH. Created with Biorender.com.

Etiology of PAH can be characterized by unique biochemical profile of particular miRNAs expressions. Usually, changes in the concentrations of many miRNAs are observed compared with healthy volunteers. In some etiologies of PAH, the bone morphogenetic protein receptor type II (*BMPR2*) gene has been found mutated (42). Product of this gene transcription, BMPR2 protein is a serine/threonine receptor kinase, which is the most important mediator of vascular remodeling in PAH. The dysregulation of this receptor kinase has been found in hereditary PAH (43, 44), and downregulated in some other etiologies of PAH (42). The downregulation of BMPR2 may be caused by the action of miRNAs, which appeared to be essential regulators in the pathogenesis of PAH, hence their potential use as biomarkers in this disease as a whole, and also in particular etiologies of PAH. MiRNA was experimentally proven to regulate the expression of BMPR2 (42). An altered expression miR-125a in lung tissue samples of experimental PAH was observed. There is a possibility of evaluating expression levels of miR-125 in order to assess hereditary PAH, since miR-125 takes part in downregulation of BMPR2, which has been seen in this particular PAH etiology. Potential role of miRNA as a regulator of BMPR2 is presented in Figure 1. Importance of BMPR2 signaling pathway in PAH pathogenesis was also proven in a recent study with sotatercept. It is a first-in-class therapeutic fusion protein that targets an imbalance in activin–growth differentiation factor and BMPR2 pathway signaling (45). Twenty four-weeks treatment with sotatercept reduced pulmonary vascular resistance among patients with pulmonary arterial hypertension who were receiving stable background therapy, including prostacyclin infusion therapy. Concordant improvements from baseline in 6-min walk distance (6MWD) and NT-proBNP levels were also observed. 6MWD is a parameter used to assess functional capacity. It is widely used in clinical trials to compare the effects of treatments (46).

### MicroRNA to define the severity of pulmonary arterial hypertension

MiRNAs could be used to evaluate the hemodynamic and clinical advancement of PAH. There are studies showing that the concentration of miRNAs in lungs of patients with PAH is different compared with samples from healthy controls (20). Correlation between hemodynamic severity of PAH and plasma concentration of specific miRNAs could be crucial in a diagnostic process and could also provide essential prognostic information (20).

#### MiR-150

In a recent study, miRNA plasma level in patients with PAH were measured. A microarray screen was performed on plasma RNA from healthy controls and patients with PAH. They identified 58 miRNAs that were dysregulated, with miR-150 being most significantly downregulated in patients with PAH (47). Quantitative polymerase chain reaction confirmed reduced miR-150 concentrations and was then used to measure miR-150 levels. Cox regression analysis confirmed miR-150 levels as a significant predictor of survival (47). This information could potentially be utilized to evaluate the severity of PAH based on plasma levels of specific miRNAs.

The source of circulating miR-150 in PAH is unknown (47). MiR-150 expression levels were significantly reduced in rats with PAH compared with control subjects. This was also accompanied by a significant reduction in *KLF2* expression, which is a critical regulator of endothelial gene expression and may reflect endothelial dysfunction and inflammation (47, 48). Importantly, microRNAs such as miR-150 mediate downstream effects of *KLF2* in endothelial cells, its expression being promoted by laminar flow and inhibited by turbulent flow and inflammatory cytokines (48).

Altered circulating miR-150 levels in PAH could affect a variety of targets contributing to the dysregulation of pulmonary arterial smooth muscle cells (PASMCs) and pulmonary arterial endothelial cells (PAECs) (47). Altogether miR-150 has a potential to be a marker of PAH severity, but it needs further investigation.

#### MiR-204

**FIGURE 2:**
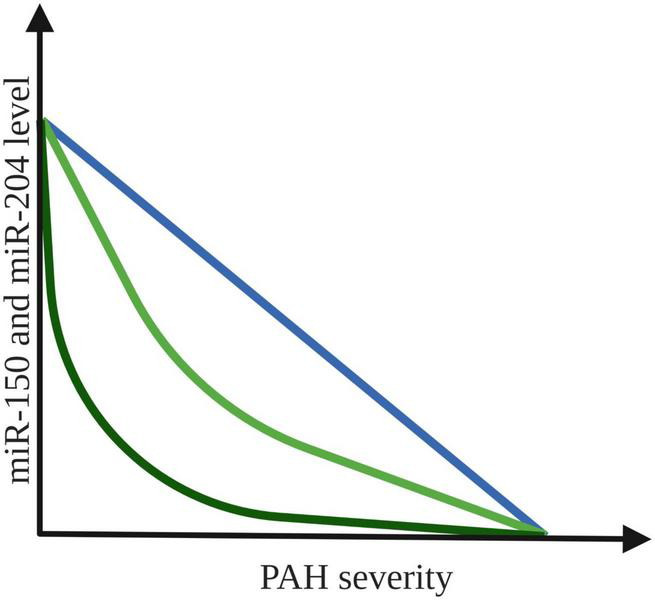
Possible correlation between expression level of miR-150 and miR-204 and PAH severity. The colored lines indicate an association between the increased PAH severity and the decrease in plasma expression of both miR-150 and miR-204 levels (31, 47). Created with Biorender.com.

Similar conclusion comes from another study, in which the expression pattern of miR-204 in lung biopsies from individuals with and without PAH was investigated. The results showed that lower concentration of miR-204 correlate with increased PAH severity (31), which could be used in clinical diagnostics in the future. On the other hand, in a study of 47 patients with PAH blood samples were collected during RHC. MiR-204 concentration increased sequentially along the pulmonary vasculature (log fold-change slope = 0.22 [95% CI = 0.06–0.37], *P* = 0.008) (30). Authors of mentioned study propose that the primary contributor to this finding of disease mediated miR-204 excretion appear to be PASMCs. Such reciprocal directions of intracellular and extracellular miRNAs concentrations are not without precedent, as aerobically exercising skeletal muscle tissue has been shown to release specific miRNAs to allow for a more efficient reduction of miRNA activity in the intracellular space of source tissue (49). The possible types of correlation between the decreased levels of both miR-150 and miR-204 and increased severity of PAH are presented in Figure 2.

The downregulation of miR-204 accounts for the upregulation of the transcription factors, such as nuclear factor of activated T cells (NFAT) and hypoxia induced factor 1α (HIF-1α) in PAH-PASMCs, which is contributing to their proliferation and resistance to apoptosis (50). In addition to that, PAH-PASMCs produce higher levels of IL-6, which by activating STAT3, contributes to further miR-204 downregulation (50). This closes the vicious circle of PAH development in some patients. Nonetheless, the implication of other pathways cannot be ruled out.

For example, increasing miR-204 level in PAH-PASMCs decreased *Src homology 2 domain containing (SHP2)*, whereas miR-204 inhibition in control PASMCs cells increased *SHP2* expression. These results suggest that *SHP2* may be the primary target of miR-204 in PAH. *SHP2* activation results in activation of STAT3, which is an activator of NFAT. As mentioned before, NFAT is important factor in proliferation and resistance to apoptosis of PAH-PASMCs. The complexity of miR-204 activity gives it the potential to became a biomarker of PAH.

#### Changes in microRNA concentration correlate with changes in echocardiography

In a study with 12 pediatric PAH patients it was shown that there are correlations of circulating miRNA concentrations with invasive hemodynamic and non-invasive echocardiographic variables. Numerous miRNAs (miR-193a-5p, miR-26a-5p, miR-331-3p, miR-29a-3p, miR-26a-5p, miR590-5p, and miR-200c-3p) positively correlated with the prognostic variables, except for those that decrease with the severity of the disease (tricuspid annular plane systolic excursion and pulmonary flow index) (51). The correlations of the miRNAs which likely exacerbate the disease (miR-423-5p and miR-99a-5p) were in the opposite direction in comparison to the likely beneficial miRNAs (51).

Furthermore, this study provided evidence that sampling site should be taken into account. MiR-193a-5p and miR-423-5p concentrations have differential trans-right-ventricle gradients (PAH patients vs. control) (51). Moreover, these concentration differences correlate with invasive hemodynamic variables. It was the first time that trans-right-ventricle and transpulmonary miRNA gradients in blood plasma were identified. It appears that both the tissue and the specific place of its collection should be taken into account (e.g., the expression of miRNA in blood from the right ventricle differs from that from pulmonary arteries). This observation also should be considered in future tests validation.

## MicroRNA as potential therapeutic targets in pulmonary arterial hypertension

MiRNA-based therapy is promising due to characteristic of miRNA, which are short length, highly conserved sequence, and pre-clinical evidence of their efficacy in human disease. Treatment based on miRNA focuses on 2 different approaches: (1) specific artificial elevation by miRmimics; or (2) lowering of selected miRNA levels, which can be achieved by anti-miRNA antisense oligomers (antagomiRs), masking, sponges, and erasers (52). *In vitro* studies and animal models, though, have produced promising results with potential for a future clinical application (53–55).

The important therapeutic targets in PAH are the PAECs, PASMCs and pulmonary adventitial fibroblasts, which excessive proliferation and resistance to apoptosis is a cornerstone in the pathogenesis of PAH (56). Because miRNAs play an important role in regulating the proliferation, differentiation and metabolism of PASMCs, they are being an promising targets in preventing these mechanisms (57). Reduction of miR-204 expression was observed in PASMCs of patients with PAH vs. healthy controls (31). It was caused by primary signal transducer and activator of transcription (STAT3) activation by circulating pro-PAH factors such as endothelin-1, platelet-derived growth factor (PDGF), and angiotensin II (which all increase at the onset of PAH) (58). It results in the sustained pro-proliferative and antiapoptotic phenotype of cultured PAH-PASMCs (31). MiR-204 mimics can reverse this effect in rats (31). It was reported that increased lung levels of miR-204 inhibited vascular remodeling by suppressing STAT3 signaling (32). As a result, the use of miR-204 mimics in rats indicated a decrease in pulmonary pressure, a decrease in the thickness of the right ventricular wall and a decrease in the thickness of the walls of the pulmonary vessels (31).

**FIGURE 3:**
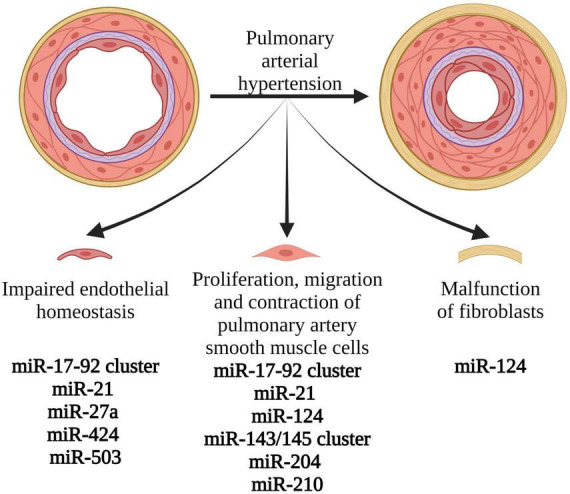
Pathophysiological mechanisms involved in the development and progression of pulmonary arterial hypertension, associated with miRNA expression changes. Modification of levels and function of the presented miRNAs might create novel therapeutic options in PAH. Created with Biorender.com.

Moreover, the effectiveness of antagomirs’ therapy has been proven in animal models with the use of antagomir to miR-17 (59). Its application in PAH mice model resulted in a reduction of pulmonary pressure and right heart hypertrophy (59). It has been suggested that these therapies have some advantages over currently available drug therapies. They are based on the action of single miRNA on multiple pathways, which cause PAH simultaneously. Therefore, they may be associated with better treatment outcomes and a lower risk of drug resistance over time (31). MiRNAs involved in the various mechanisms of PAH development, which might be targets of future PAH therapy, are presented in Figure 3.

## Challenges in microRNA analysis and interpretation in pulmonary arterial hypertension

The huge development in the use of miRNAs as PAH biomarkers is not without limitations. In the last 15 years, as the new miRNAs were discovered in animal and human PAH models, there have also been comments on the challenges associated with these studies and the use of miRNAs in the analysis and interpretation of PAH. Such limitations include: (i.) various mechanisms affecting mRNA expression; (ii.) miRNA differences between experimental animal models and humans; (iii.) differences in the pathogenesis of PAH in humans (3, 60, 61).

### Various mechanisms affecting mRNA expression

In the perfect situation, level of mRNA is constant and the level of the protein produced by it is lowered or increased due to a disturbed level of miRNA (61). However, various mechanisms influence mRNA expression. The long non-coding RNAs (lncRNA), which plays a role not only in regulating post-transcriptional mechanisms, such as miRNA, but also transcriptional mechanisms, may be also responsible for changes in mRNA expression (3, 16, 62, 63). The role of lncRNA has been demonstrated, *inter alia*, in the pathogenesis of PAH resulting from PASMC and PAEC dysfunction (3, 61). Hence, in cases of PAH where the mRNA level is normal and the level of the protein produced from this mRNA is abnormal, the miRNA biomarkers may be partially insensitive due to overlapping factors, such as lncRNA (64). LncRNAs are >200 base pairs in length and are abundantly present in the genome. Almost 27,000 lncRNAs have been identified in the human genome already (65). They are differentially expressed in key pulmonary vascular cells such as PAECs and PASMCs, which regulate a range of cellular and biological processes and contribute to pulmonary vascular remodeling and PAH pathogenesis (64). Summary of lncRNA involved in PAH is presented in Table 2.

**TABLE 2:** LncRNA involved in pulmonary arterial hypertension.

LncRNA	Subcellular localization	Experimental models used	Expression in PAH	Key findings	Mechanisms	References
TYKRIL	Nucleus and cytoplasm	Hypoxia-induced PAH in human PASMCs	↑ in PASMC	Enhances proliferation, inhibits apoptosis of human PASMCs under hypoxia.	p53/PDGFRβ axis	(105)
PAXIP1-AS1	Both nucleus and cytoplasm	Small pulmonary arteries of IPAH patients	↑ IPAH-PASMCs and pulmonary arteries	Inhibition promotes apoptosis and inhibits PASMC proliferation and migration	Paxillin	(106)
HOXA-AS3	–	Hypoxia-induced PAH in human PASMCs	↑ in lung, PASMC	Modulates cell cycle and enhance proliferation in PASMC	HOXA3	(107)
MALAT1	–	–	↑ in pulmonary arteries, PASMC	Accelerates cell cycle progression as well as pulmonary vascular remodeling	hsa-miR-124-3p.1/KLF5	(108)
MALAT1	–	Hypoxia-induced PAH in human PASMCs	↑ in plasma and hypoxic human PASMCs	Knockdown reduces human PASMCs proliferation and migration while promotes their apoptosis	miR-503/TLR4 Axis	(109)
UCA1	Predominantly in cytoplasm	Hypoxia-induced PAH in human PASMCs	↑ in PASMC	Promotes proliferation and inhibits apoptosis in PASMC	hnRNP I	(110)
LincRNA-COX2	–	Hypoxia-induced PAH in PASMCs	↑ in blood, PASMCs	Promotes PASMCs proliferation	LincRNA-COX2/miR-let-7a/STAT3 axis	(111)
PAHRF	–	Hypoxia-induced PAH in PASMC	↓ in PAs of PAH patients and hypoxic PASMC	Downregulation of PAHRF promotes PASMC proliferation and inhibits apoptosis	PAHR/miR-23a-3p/MST1 axis	(112)

### MicroRNA differences between experimental animal models and humans

One of the challenges regarding the use of miRNAs as biomarkers of human diseases is deriving conclusions from studies conducted in animal models (60). Although these models show similar miRNA expression for healthy animals as healthy humans, miRNA production for diseased individuals differs in animals and humans (60). These differences are due to different pathophysiological mechanisms of PAH in animals, which are also phenotypically different from human PAH in many aspects (60). However, animal studies are necessary, because the small number of PAH patients makes it difficult to develop cohort studies. In many cases, there is also a need to perform invasive procedures (60). The triggers of PAH used in animal models include, but are not only limited to, administration of monocrotaline or chronic hypoxia (60). The challenge is therefore to find the PAH trigger that most closely matches the cellular and molecular changes present in human PAH.

### Differences in the pathogenesis of pulmonary arterial hypertension in humans

Another challenge for the use of miRNA as a biomarker is the likely existence of multiple PAH etiologies with different miRNA expression in one patient (3, 66). These etiologies differ in the number of inflammatory and hypoxic stimuli leading to the formation of PAH and causing different expression of miRNAs (42). A suggestion for the existence of different etiologies of PAH in patients is that the animal models of PAH which are otherwise induced, show different miRNA expression (3, 27). Thus, the usefulness of many biomarkers may depend on the etiological factors of PAH.

Furthermore, miRNA expression may also depend on the degree of progression of PAH in the patient. There are evidences, that it depends on the development of right ventricular (RV) hypertrophy or RV failure (3). Some studies suggested that different miRNAs may be detected if there is the presence of RV hypertrophy or failure (28, 29). Thus, the detection of miRNAs, which are produced by dysfunctional heart muscle, should take this phenomenon into account.

### Analytical considerations in microRNA quantification

There are numerous factors influencing the accuracy of miRNA analyzes. A critical evaluation of such factors is of paramount importance in order to avoid misleading results and to allow the comparability of different analyzes. For example, in RNA isolation methods, maximum efficiency, reproducibility and reliability are of great importance, because, e.g., even minimal co-extraction of inhibitory factors can significantly affect the measurement results. Various detection methods (high-throughput sequencing, real-time PCR, microarrays) are used to quantify miRNA levels. Their application should be determined for specific indications to minimize variation between studies. Standardization method should be standardized and reproducible isolation and detection protocols applied; otherwise, comparability is much more difficult (67). In addition to these analytical aspects, there are parameters influencing the levels of miRNAs in the analyzed biomaterial. Drugs such as statins, antiplatelet drugs and heparin can have a confusing effect on miRNA measurements. For example, antiplatelet drugs reduce the amount of freely circulating thrombocyte-derived miRNAs, and heparin influences the polymerase chain reaction (68). Finally, there are differences in the results of miRNA quantification between cellular and extracellular sources of biomaterial (69).

In a study of 2,391 individuals expression of miRNAs measured by high-throughput RT-qPCR isolated from both whole blood and were compared. There was only modest association between cellular and extracellular miRNA expression in this large study sample (69). These results demonstrate that miRNA expression from cellular vs. acellular sources may be divergent. MiRNA concentration from different human sources (e.g., whole blood, plasma) should not be used interchangeably as biomarkers of disease (69).

## Steps to take before clinical applicability of microRNA in pulmonary arterial hypertension

Together with the first discoveries in animals related to the role of miRNA in the development of PAH, it was known that these findings should be put into clinical practice as soon as possible. At this point, two possibilities for the future use of miRNAs in PAH patients emerge: (i) miRNAs as biomarkers and (ii) miRNAs as therapeutic targets (3).

### MicroRNAs as biomarkers

The first option is to use miRNA as a diagnostic marker (3, 20). So far, it has not been possible to find an ideal biomarker for PAH, i.e., one that is non-invasive, easy of measure, cost-effective, and has high sensitivity and specificity (70, 71). There is a great hope in finding such a marker among the miRNAs (56). For this purpose, the problem of different fragments and levels of miRNAs in experimental studies caused by the lack of their standardization should be solved (3, 56). It is caused by the lack of systematization in the procedure of taking, storing and processing samples; lack of standardization in the methods of miRNAs diagnosing and lack of indications for their use; lack of standardization in miRNA quantification protocols; rare sampling in the studies and lack of described concomitant disease symptoms (3, 56, 70, 72). After solving the above problems, it would be possible to summarize the results of many different studies and use the obtained results to create studies on a large samples (56). These studies would justify the use of specific miRNAs as biomarkers. Machine learning might be useful for selection of the most promising miRNAs. Machine learning as a field has progressively improved our ability to find relevant features in large and high-dimensional data sets collected from genomic studies (73). Supervised machine learning methods have been used successfully to develop classifiers for disease diagnosis, as well as to identify potential disease biomarkers (74). In a recently published study it was shown that using a consensus of four different supervised machine learning feature selection techniques can help us to identify miRNAs associated with PAH (72). MiR-187-5p and miR-636 were selected as a candidate biomarkers that might be associated with PAH progression, however this method requires further studies and validation (72).

As mentioned earlier, miRNA concentrations in RV hypertrophy and RV failure vary (27, 28). This suggests that during the progression of RV overload caused by, *inter alia*, PAH there are some changes in miRNA expression. More research is needed to see if we can predict PAH progression from changes in miRNA levels even before structural changes occur, especially in patients with high risk of developing PAH.

The final step of identification of perfect biomarker would be to perform a cost-effectiveness analysis to determine whether the ratio of new information obtained from such a research and its price is satisfactory to use such a miRNA for the diagnosis of PAH (56).

### MicroRNA and its therapeutic role

The future prospects of the miRNA are also apparent in their use in the treatment of PAH. Mainly, such therapy would be based on the use of miRNA analogs to increase the amount of miRNA that is decreased in PAH, or miRNA inhibitors to decrease the amount of miRNA whose expression is increased (20, 56). So far, the experiments in animal models show promising results (3). Before they can be used clinically, however, some problems have to be solved. These include: low drug stability, off-target effect and drug delivery to the selected tissue (3, 20, 56, 75).

#### Stability of microRNA-based drugs

MiRNAs are small particles, which are degraded *in vivo* by nucleases. Apart from chemical modifications to improve their stability, miRNA based therapeutics are encoded as prodrugs, in order to protect the active substance from the degradation mechanisms of exogenous genetic material that exist in eukaryotic cells (76). To improve stability, resist degradation by RNA nucleases and slow their removal *in vivo* by the liver there are needed some structural modifications. The most common chemical modifications alter the internucleotide connections or replace backbone phosphodiester for phosphorothioate, boranophosphate or peptide bonds (77).

MiRNAs uptake can be also improved by decorating miRNAs with receptor-mediated endocytosis pathways. On the other hand, cell uptake can be improved by incorporating, at the 3′ end, lipids or cholesterol which increases the permeability through the cellular lipid bilayer (77). The strategies to chemically modify oligonucleotides have been the subject of many recent reviews and debates about their influence on the pharmacological properties or miRNA-based therapies since often they reduce their efficiency (78, 79).

#### The off-target effect

The off-target effect is associated with the miRNA effect on various tissues, including healthy ones, disrupting cellular pathways unrelated to disease pathogenesis, and inducing an immune response, resulting in the toxic effects of these drugs (3, 20). To reduce the off-target effect it is crucial to choose optimal delivery method. In rats, intranasal aerosolization (IN-A), intratracheal aerosolization with (IT-AV) or without ventilator assistance (IT-A), and intratracheal liquid instillation (IT-L) were compared (80). Intravenous (IV; via jugular vein), intraperitoneal (IP) and subcutaneous (SC) delivery served as controls. Relative levels of cellular miR-39 were quantified by RT-qPCR. At 2 h post-delivery, IT-L showed the highest lung mimic level, which was significantly higher than levels achieved by all other methods (from ∼10- to 10,000-fold, *p* < 0.05) (80). All lung-targeted strategies showed lung-selective mimic uptake, with mimic levels 10- to 100-fold lower in heart and 100- to 10,000-fold lower in liver, kidney and spleen (80). In contrast, IV, SC, and IP routes showed comparable or higher mimic levels in non-pulmonary tissues. Altogether the choice of delivery strategy could have a significant impact on potential therapeutic outcomes of miRNA-based drug candidates. To sum up, intratracheal administration of a liquid formulation of miRNA mimic provided the highest lung-specific delivery and appears to be the safest therapeutic delivery method in terms of off-target effect.

#### Efficiency and vectors

MiRNA-based therapy suffer from low stability and efficiency (81). To prevent these adversities they require chemical modifications (82, 83) or conjugation/encapsulation in different kinds of nanovectors such as lentivirus, polymeric nanoparticles, or exosomes (84–86).

Virus-like nanovectors (e.g., adenovirus, lentivirus) are used for delivering miRNAs because they dispense miRNAs more efficiently than other carriers (87, 88). On the other hand they can induce mutagenic effects causing cancer and undesired immune responses. These undesired effects limits their clinical application, so their real translation into clinics is controversial (89). However, their exact composition is uncontrolled. They contain active molecules with unknown effects for the organism.

In contrast, extracellular vesicles (EVs) as drug delivery carriers are in ongoing clinical trials aiming to translate them from basic research to clinics (77). They are lipidic bilayer vesicles naturally released by cells which size varies from 10 to 10,000 nm. The main advantage of these nanovectors is that they can be obtained from patients avoiding toxicity (90). EVs are often polydispersed in size and can be further functionalized with nanoparticles, for example for therapy (91). They are currently being used as successful drug delivery systems for miRNAs into particular cells, such as hepatocytes or macrophages (92).

Another possible vector are synthetic nanovectors. They can be finely designed in the nanoscale and manufactured in a large scale (93). Encapsulation of miRNAs has been mainly carried out using polymers and lipids (94, 95). Often the driving force for the entrapment of negatively charged miRNAs is the electrostatic attraction with cationic lipids or polymers (77). Physical caging is also another efficient way to entrap miRNAs in empty cavities (77). Lipofectamine is considered the “gold standard” for nucleic acid transfection. It is a lipid-based nanovector composed of positively charged cationic lipids. Often it is called a “liposome” (77). However, the use of permanently charged cationic lipids can induce cellular and inflammatory toxicity, fast plasma clearance, aggregation, and accumulation in lungs, liver, and spleen (77). In this review, we only give a brief summary of possible vectors for miRNA-based therapy.

Studies of the miRNAs and their therapeutic role would be facilitated by the discovery of animals models, that phenotypically copy PAH in humans, and by finding an antidote to the potential adverse effects of these drugs (3, 56). After resolving the above-mentioned issues, the next steps should be taken in regard to the risk of miRNA and other drugs interferences, standardizing the drugs used and their doses in relation to comorbidities, age and sex of patients, as well as performing studies on a larger number of model animals, and clinical phase 1, 2, and 3 studies, which so far have not been undertaken for miRNAs in PAH (3, 56).

## Conclusion

The role of miRNAs in the pathogenesis of PAH cannot be overlooked. With the still developing biotechnology and knowledge about PAH, new miRNAs involved in the pathogenesis of this disease are found every day. From this reason, the range of miRNAs that may be useful in the diagnosis and treatment of PAH is constantly extending. So far, miR-29, miR-124, miR-140, and miR-204 are the most promising miRNA-bases biomarkers and/or therapeutic targets in patients with PAH. To take a step forward in the clinical application of these miRNAs, both more experimental studies and clinical trials with bigger cohorts of are needed to evaluate these miRNAs as biomarkers and therapeutic targets. However, it should be considered whether research concentrated on quantifying several miRNAs at the same time as cluster-biomarker and therapy aimed at group of miRNAs at the same time would not be a better solution in cost-quality ratio than trial on single miRNA. In addition, the difficulties associated with studies on miRNAs and using miRNAs in clinic should be addressed when designing miRNA studies.

## Author contributions

SR, BP, NC, and AG: conceptualization and writing—original draft preparation. SR, BP, NC, and CE: methodology. CE, MP, ŁS, KF, MJ, TM, MG, and MK: writing—review and editing. AG: visualization and supervision. SR and AG: project administration. All authors have read and agreed to the published version of the manuscript.
